# Contralateral C7 transfer to lower trunk via a subcutaneous tunnel across the anterior surface of the chest and neck for total brachial plexus root avulsion: a cadaveric study

**DOI:** 10.1186/s13018-019-1068-2

**Published:** 2019-01-23

**Authors:** Juntao Feng, Tao Wang, Pengbo Luo

**Affiliations:** 1 Department of Orthopedics, Shuguang Hospital, Shanghai University of Traditional Chinese Medicine, No. 185, Pu An Road, Shanghai, China; 2 Department of Hand Surgery, Huashan Hospital, Fudan University, 12 Wulumuqi Zhong Road, Shanghai, China; 3 Shanghai Key Laboratory of Peripheral Nerve and Microsurgery, Shanghai, China; 4 Department of Orthopaedic Surgery, Shanghai Jiaotong University Affiliated Sixth People’s Hospital, No. 600 Yishan Road, Shanghai, 200233 China

**Keywords:** Brachial plexus, Cadaver, Nerve transfer, Nerve regeneration, Thorax

## Abstract

**Background:**

Restoration of hand function after total brachial plexus root avulsion (tBPRA) is a difficult problem in surgical management. A new modified approach in repairing tBPRA is to use a subcutaneous tunnel across the anterior surface of the chest and neck, and then transfer the contralateral C7 root (cC7) to the lower trunk. However, the anatomical details of this method have not yet been fully described and assessed. The objective of this study was to quantitatively describe the nerve transfer using a cadaveric surgical simulation.

**Materials and methods:**

Brachial plexuses were dissected from 12 adult cadavers, producing 24 sides of brachial plexuses for nerve transfer experiments. We performed simulated cC7 transfers to the lower trunk via a subcutaneous tunnel across the anterior surface of the chest and neck. Measurements of the nerves were made and transfers quantitatively documented.

**Results:**

With the affected shoulder and arm in a neutral position, cC7 and C8-T1 could be sutured directly together in 75% of the cadavers. A nerve graft length of 4.6 ± 1.18 cm was needed to bridge the gap in the remaining cadavers. For cadavers where distal cC7 was directly connected with the lower trunk, 54.17% could be sutured, and an average nerve graft length of 3.9 cm was needed in the remains.

**Conclusions:**

For surgical management of total tBPRA, transfer of the cC7 nerve to the C8-T1 or lower trunk via a subcutaneous tunnel across the chest and neck will likely be superior to a conventional cC7 root transfer in the clinic. This approach shortens the nerve graft needed and nerve regeneration distance, decreases the number of neurorrhaphy sites, and makes full use of the donor nerves, which may benefit hand flexion restoration.

## Background

Total brachial plexus root avulsion (tBPRA) is one of the most devastating trauma injuries, causing total paralysis and loss of sensation in the affected limb. Surgical nerve transfer is the primary choice for restoring motor and sensory function. For over 30 years, the contralateral C7 (cC7) root transfer method has been used to surgically repair tBPRA, especially when other donor nerves are in short supply [1]. However, restoration of hand function using this traditional approach remains a technical and functional challenge. Using a long graft to bridge the gap between the donor C7 root and the recipient nerve is disadvantageous for encouraging early re-innervation of the recipient nerve [2]. Hence, shortening the length of the graft would be beneficial for functional recovery [3]. A modified technique in which the cC7 root was transferred to the lower trunk or C5/6 via a prespinal route with direct neurorrhaphy has been reported to shorten the regeneration distance in tBPRA cases [4, 5]. One disadvantage of this modified cC7 transfer via the prespinal and retroesophageal route is that it requires a dissection in the cervical region, which is anatomically complex [5]. Furthermore, re-exposing the nerve connection would be difficult when nerves in this region could have been ruptured during tBPRA. Thus, we designed an alternative method for tBPRA repair that mitigates these problems.

To reduce adverse effects, simplify the procedure, and improve functional outcome, we developed a cC7 nerve transfer via a subcutaneous tunnel across the chest and neck to repair connections to the C8-T1 or lower trunk. We successfully applied this alternative in four patients previously [6]. Objective evidence of functional recovery using this new method was obtained in these patients at a 26- to 38-month post-operation assessment; our results on these four patients suggest that this approach is innovative, feasible, and an effective surgical procedure for tBPRA repair. It was found that mobilization of the avulsed lower trunk made it possible to reach the suprasternal notch [7]. Anatomical and biomechanical experiments indicated that preganglionic lesions were mainly produced in C8 and T1, causing the avulsion of nerve roots from the spinal cords, and making them unavailable to be repaired without resection of the nerve roots [8, 9]. Thus, more anatomical evidence with quantitation would bolster our promising clinical results and facilitate its adoption. Using a cadaveric surgical simulation in the present study, we comprehensively described and quantified the anatomical details of donor C7 and recipient nerves with use of the cC7 root transfer via this modified approach.

## Methods

### Specimens

Twenty-four brachial plexuses from 12 formalin-fixed cadavers were analyzed. There were seven male and five female adults.

### Dissection procedure

**Fig. 1:**
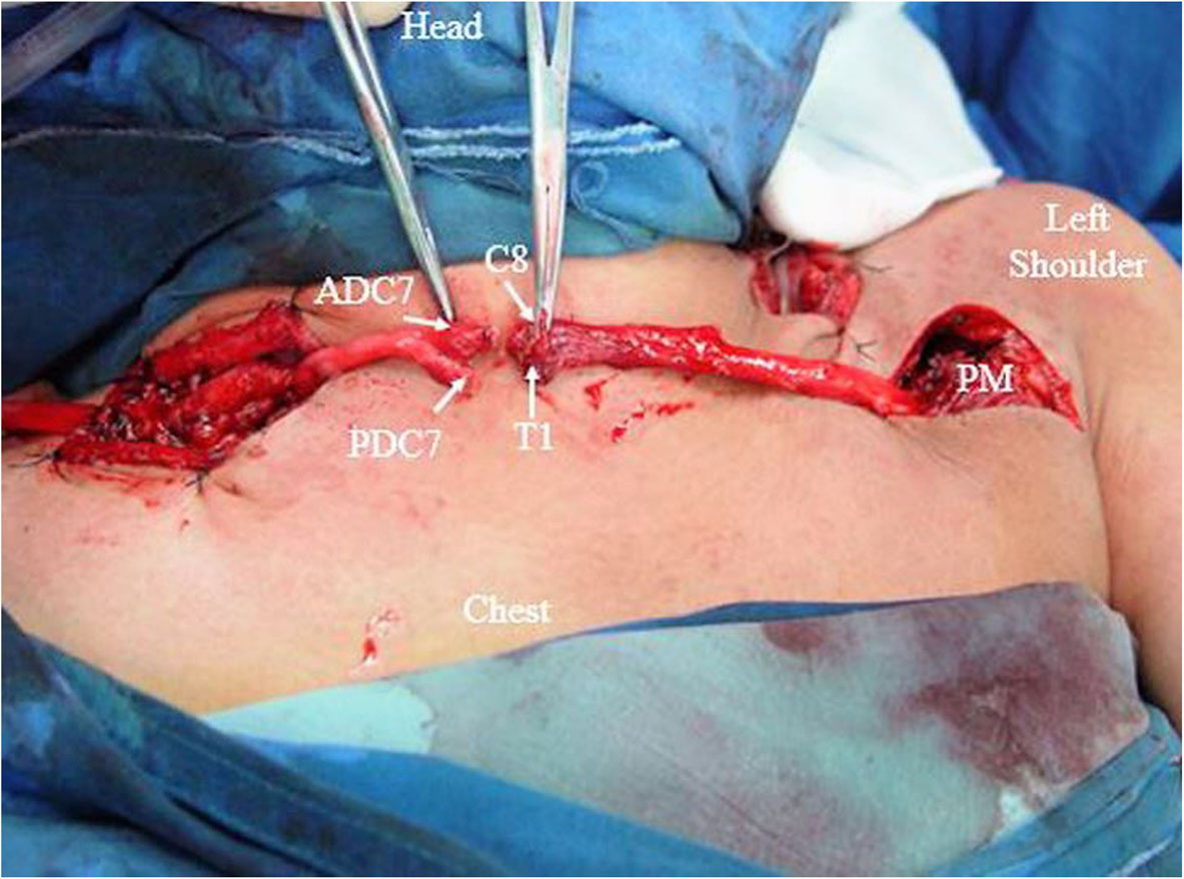
Intra-operation photograph of a clinical practice, illustrating the modified cC7 nerve transfer procedure. Microdissection was carried out to isolate the divisions of the right (donor side) C7 as distally as possible before joining the corresponding cords. Next, the anterior and posterior divisions of the C7 (ADC7, PDC7) were disconnected beneath the sternocleidomastoids and traced to the contralateral side. The C8 and T1 roots on the left (recipient side) were disconnected at the level of the intervertebral foramen and pulled distally beneath the clavicle to the proximal part of the pectoralis major (PM). After the nerve suture site was determined, another transverse incision on the anterior middle neck was made, followed by direct neurorrhaphy. The patient was a 48-year-old male

To facilitate presentation and understanding of our modified cC7 transfer procedure, we provide an intra-operation image (Fig. 1).

#### Exposure of brachial plexus

**Fig. 2:**
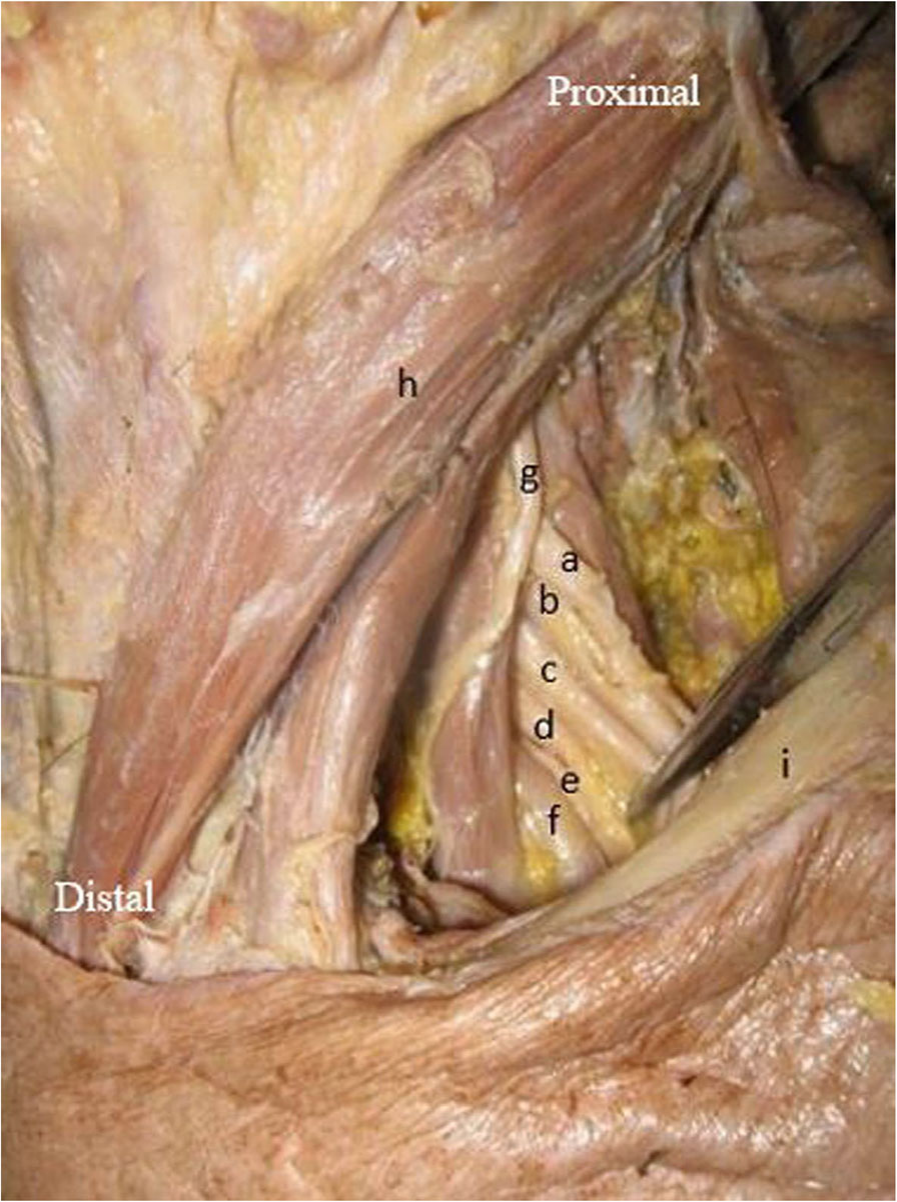
Gross anterior view of a formalin-fixed cadaver (54-year-old male). Anatomy of left brachial plexus in supraclavicular region. The left C7 served as the donor nerve in this case. (a) C5, (b) C6, (c) C7, (d) C8, (e) T1, (f) subclavian artery, (g) phrenic nerve, (h) sternocleidomastoid muscle, (i) clavicle

**Fig. 3:**
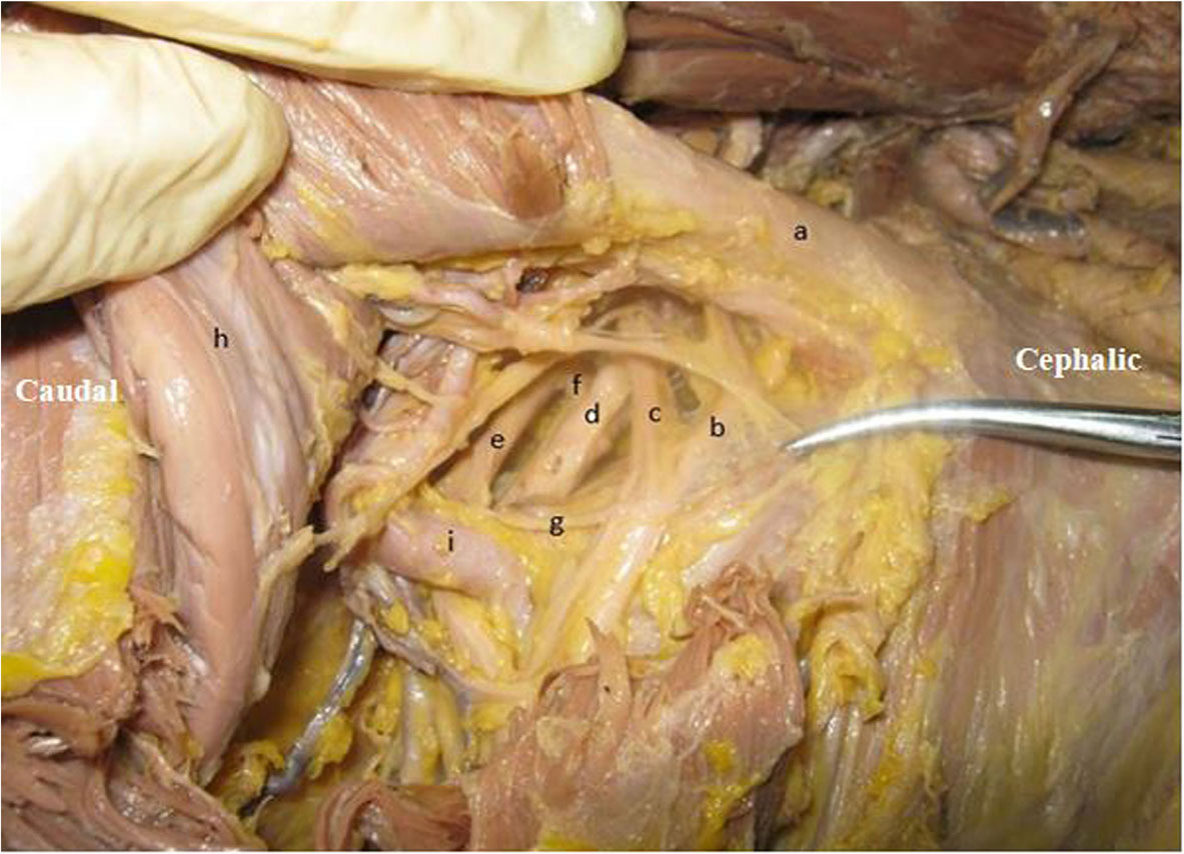
Gross anterior view. Anatomy of left brachial plexus in the infraclavicular region. The left C7 served as the donor nerve in this case (54-year-old male). (a) clavicle, (b) anterior division of upper trunk, (c) anterior division of C7, (d) posterior division of C7, (e) medial cord, (f) posterior division of lower trunk, (g) lateral pectoral nerve, (h) pectoralis minor, (i) axillary artery

The cadaver was placed in a supine position, and the shoulder was abducted between 45° and 90°. After the skin of the chest was removed, the brachial plexus was explored above and below the clavicle. The clavicle was not osteotomized. After the deltoid-pectoral groove was explored, the pectoralis minor muscle was cut from its end, and the medial cord, lateral cord, posterior cord, anterior division of C7 (ADC7), and posterior division of C7 (PDC7) were exposed. After the nerves were identified (Figs. 2 and 3), distance measurements were made bilaterally.

### Anatomical dissection and measurements of C7

**Fig. 4:**
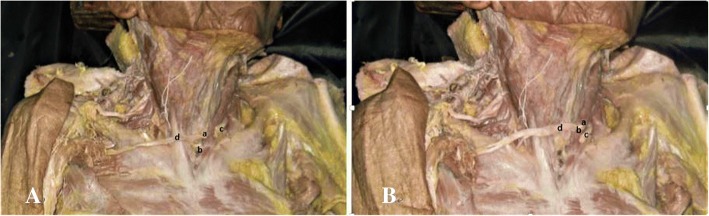
Normal in situ length of the anterior and posterior divisions of C7 before micro-elongation dissection (**A**, (c)). After microdissection (**B**), the distal ends of C7 were disconnected beneath the sternocleidomastoids and traced contralaterally to the sternoclavicular joint. We then measured the distance between the anterior midline and the C7 ends. (a) C8, (b) T1, (c) C7 ends, (d) lower trunk

The C7 root was dissected proximally to the intervertebral foramen and distally to the ends of its anterior and posterior divisions. The length from the C7 intervertebral foramen to the end of the ADC7 and PDC7 was measured. To facilitate this microdissection, a 10% acetic acid solution was dripped onto the nerve surface, enabling the ADC7 and PDC7 to separate and micro-elongate without damage. After the epineuria at the junctions of the division cords were separated, microdissection was carried out to isolate the two divisions as distally as possible before joining the corresponding cords. The micro-elongated lengths of ADC7 and PDC7 were then measured again. Next, the ends of C7 were disconnected beneath the sternocleidomastoids and traced contralaterally to the sternoclavicular joint so that we could measure the distance between the anterior midline and the C7 ends. When the ends crossed the anterior midline, we assigned the distance to be a positive number, and when the nerve ends did not cross the anterior midline, we assigned the distance to be a negative number (Fig. 4).

### Anatomical dissection and measurements of C8 and T1

**Fig. 5:**
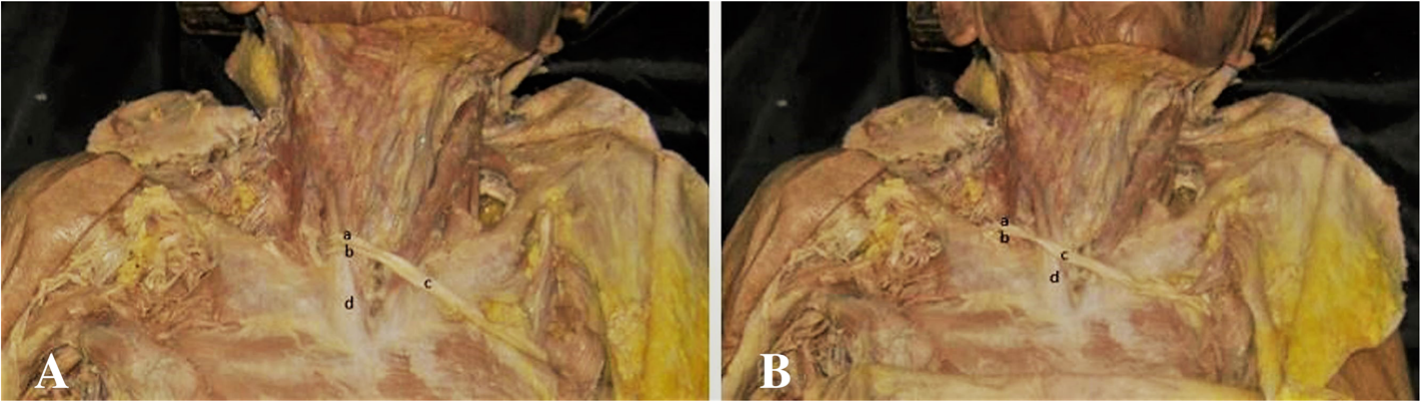
C8-T1 were pulled contralaterally to C7 across the anterior midline with the shoulder in a neutral position (**A**), and with the shoulder in 10° of anterior flexion and in 30° of adduction (**B**). (a) C8, (b) T1, (c) lower trunk, (d) sternocleidomastoid muscle

C8 and T1 were dissected proximally to the intervertebral foramen, distally to the ends of the medial and posterior divisions, then to the end of the pectoralis major muscle along the median nerve and ulnar nerve. C8 and T1 roots were disconnected at the level of the intervertebral foramen and pulled distally to the infraclavicular area after the posterior division of the lower trunk. We then severed the lateral head of the median nerve, the medial pectoral nerve, medial brachial cutaneous nerve, and medial antebrachial cutaneous nerve. The shoulder/arm of the recipient upper extremity was put in a neutral position. The distal stumps of C8 and T1 were pulled contralaterally to the cC7 via a subcutaneous tunnel proximal to the pectoralis major muscle. The distance between the anterior midline and the C8-T1 ends was then measured. When the nerve ends crossed the anterior midline, we assigned the distance as a positive number, and when the nerve ends did not cross the anterior midline, we assigned the distance as a negative number (Fig. 5).

### Nerve regeneration distance in cC7 transfer

For comparison, we performed a conventional cC7 root transfer in cadaveric simulation to estimate the length of the ulnar nerve graft needed. The ulnar nerve was harvested proximally from the level of the wrist to the upper arm and pulled contralaterally to the cC7 root via a subcutaneous tunnel. The shortest distance between the medial humeral condyle and the cC7 root along the ulnar nerve was calculated as the nerve regeneration distance.

For the modified cC7 transfer described in this study, the nerve regeneration distance was measured from the C8-T1 ends to the medial humeral condyle of the same side.

### Histological processing

We harvested fresh cadaveric C7, ADC7, PDC7, C8, and T1 nerves; the lower trunk of the brachial plexus; and the ulnar nerve. The length of these nerves had an additional 1 cm on both ends. These were fixed in 10% formalin for 24 h, washed, dehydrated in serial concentrations of ethanol, and embedded in olefin for sectioning. We used a manual rotary microtome to cut 5-mm-thick transverse sections. Sections were mounted onto glass slides and stained with hematoxylin and eosin (HE) and coverslipped. We examined the stained sections with light microscopy using a Leica DWLB2 microscope. Digital image analysis software (Version 11.0; IMT i-Solution Inc. Vancouver, BC, Canada) was used to measure the diameter and the cross-sectional area of the sampled nerve at a magnification of × 100 and calculate the presence of myelinated axons at a magnification of × 200.

### Statistical analysis

SPSS 11.5 software (SPSS, Chicago, IL, USA) was used for statistical analysis. All the parameters were expressed as means ± standard deviation (SD) for each group. One-way analysis of variance was used to evaluate significant differences between groups, and statistical significance was set at *p* < 0.05.

## Results

A total of 24 brachial plexuses in 12 cadavers were completely preserved. In most of the cases (20/24), the length of the ADC7 was longer than that of the PDC7. Compared with a conventional cC7 transfer, the nerve regeneration distance of our modified method was significantly shorter (44.8 ± 4.1 cm vs. 35.3 ± 2.9 cm).

### Length of the sampled nerves

**Fig. 6:**
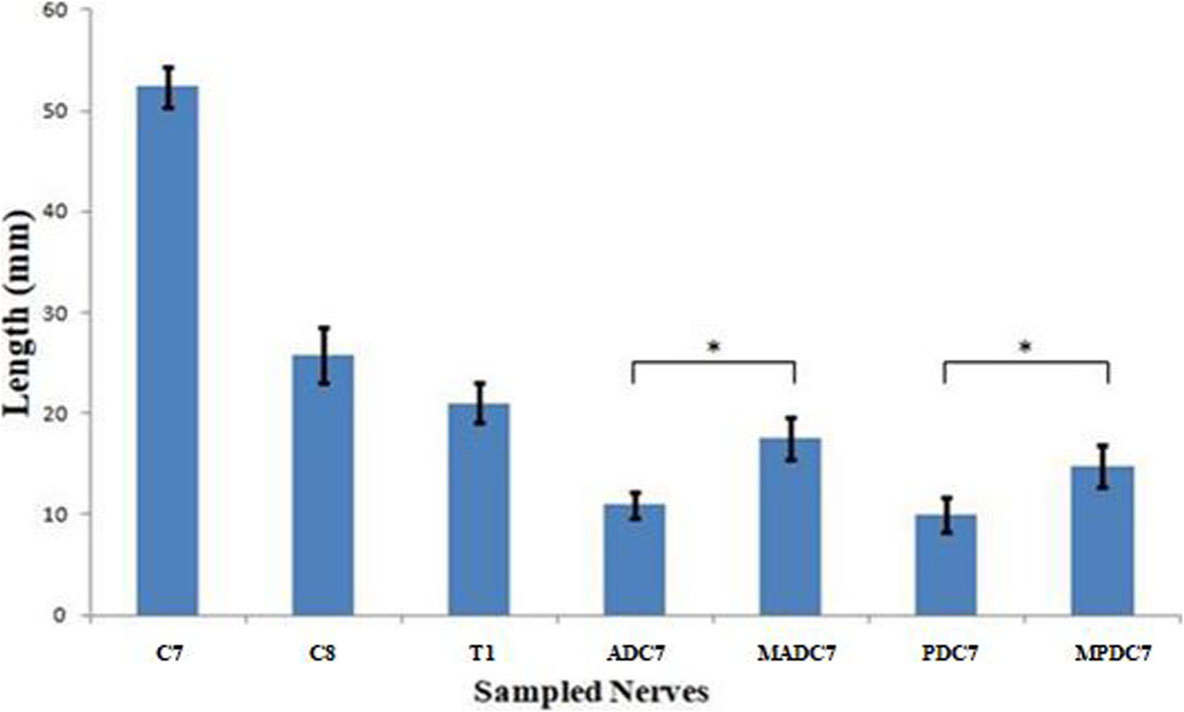
Average lengths of the sampled nerves. ADC7, anterior division of C7; MADC7, micro-elongated anterior division of C7; PDC7, posterior division of C7; MPDC7, micro-elongated posterior division of C7. *After micro-elongation dissection, nerve lengths of ADC7, and PDC7 were significantly longer (*p* < 0.05). Error bars, SD

The lengths of C7, C8, T1 roots, and divisions of C7 were similar in both male and female specimens and on both sides (gender data not shown). The lengths of ADC7 and PDC7 were significantly longer after micro-elongation dissection (Fig. 6).

### cC7 transfer to the C8-T1 and lower trunk

The distance between C8-T1 and the anterior midline was 48.55 ± 4.96 mm with the shoulder/arm in a neutral position. The distance between the lower trunk and the anterior midline was − 6.22 ± 5.50 mm. cC7 could be directly placed into the C8-T1 in 75% (18/24) of the cadaver sides. In these cases, the coaptations were accomplished without tension. For the remaining sides, where cC7 could not directly contact the C8-T1 ends, the nerve gap was 4.6 ± 1.18 cm.

cC7 could be coapted with the lower trunk in only 28.83% of the cadavers when the shoulder was in a neutral position. The nerve gap was 4.37 cm (4.37 ± 1.02 cm), on average, for the remaining ones.

### ADC7 and PDC7 transfer to the C8-T1

Our investigation revealed that in cadaveric simulation, ADC7 and PDC7 could reach the opposite C8-T1 successfully in 75% of the cases when the shoulder is in a neutral position, either before or after micro-elongation of ADC7 and PDC7. Moreover, the nerve gap was no greater than 4.8 (range, 2.13–4.80) cm for the other 25% of cadavers.

### ADC7 and PDC7 transfer to the lower trunk

ADC7 and PDC7 could be directly coapted with the lower trunk in 54.17% of the cadavers when the shoulder/arm was in a neutral position, and the nerve gap was 3.90 cm, on average, in the remaining ones. After micro-elongation dissection, the percentage of direct neurorrhaphy increased from 54.17 to 66.70%.

### Histomorphometric analysis of dissected nerves

In histological transverse sections, the diameter of C8 was larger than that of ADC7 (*p* < 0.001), and the diameter of T1 was larger than that of PDC7 (*p* = 0.024). The average number of axons in C7 was much smaller than that in C8-T1 (43,327 ± 2514 vs. 67,953 ± 4720). Also, the cross-sectional area of C7 was less than that of C8-T1 (*p* = 0.001). The number of ADC7 and PDC7 axons was comparable to that of T1 (*p* > 0.05), but C8 had significantly fewer than ADC7 and PDC7 (*p* < 0.001 and *p* = 0.007, respectively).

## Discussion

A two-stage cC7 nerve root transfer is commonly indicated for tBPRA or lower plexus root avulsion. Since 1986, the typical approach is to do the transfer through a subcutaneous route [10]. In the first stage of this standard method, C7 elongation is achieved via a pedicled reverse vascularized ulnar nerve. The second stage involves transection of the ulnar nerve at the axilla of the affected limb and connection of the elongated C7 to the selected paralytic nerves. Free or vascularized sural nerve grafts are also commonly performed via the same route. Usually, when a normal cC7 root is transferred to the median nerve of the affected limb to restore wrist flexion, finger flexion, and hand sensation, a nerve graft of over 30 cm is needed to bridge the gap in adults [11]. Various reports showed that flexion restoration after transfer of the cC7 root to the median nerve was far from what could be expected [2, 11–13]. Why might this be?

It is thought that two major factors leading to poor restoration of hand function using the traditional C7 transfer method are the long distance between the cC7 root and the target muscle and an insufficient number of donor myelinated fibers to match the recipient nerves [2]. Hence, in attempts to achieve better recovery, the cC7 transfer has been modified to shorten the gap that needs to be bridged. For instance, in a follow-up study of over 5 years, Yu et al. [14] transferred cC7 to the median nerve and ulnar nerve by shortening the upper arm. Wrist motion and partial hand function were restored, but the shortened arm was undesirable in shape and function.

Mcguiness and Kay [15] first reported a case of repairing obstetrical brachial plexus palsy, using a cC7 root transfer via a prespinal route. The nerve graft was passed through the retroesophageal space, which was thought to be a shorter distance for the graft. Validity of this procedure was verified and modified by other surgeons in the following years [4, 5, 16]. The cC7 root has been used to repair the upper trunk or the infraclavicular lateral cord and posterior cord of the injured side via a prespinal and retroesophageal route, producing a satisfactory clinical outcome [5]. The nerve graft was 6.25 ± 0.35 cm long for repairing the supraclavicular brachial plexus and 8.56 ± 0.45 cm long for repairing the infraclavicular brachial plexus [5]. They harvested 29 cm of the sural nerve and 15 cm of the superficial radial nerve for grafting.

Wang et al. [4] carried out a modified cC7 root transfer to neurotize the upper trunk and C5/C6 nerve roots in a series of 41 patients with tBPRA. A sural nerve graft with a mean length of 6.9 ± 1.9 cm was needed in these cases. The outcome was superior to that using a conventional cC7 root transfer for repairing the musculocutaneous nerve through the subcutaneous tunnel around the chest and anterior cervical.

Since the anatomy in the cervical region is rather complex, for our first attempts at cC7 transfer, we introduced a simple modified subcutaneous approach and applied it successfully, resulting in promising hand function recovery in four patients [6]. In the present study, anatomical results using cadaveric simulation further demonstrated the advantages of using this approach. First, direct neurorrhaphy of cC7 and C8T1 could be achieved in 75% of the cadavers. Second, we found that only a maximum length of 4.8 cm of graft was needed in indirect repair cases, which is nearly one-fourth the mean length required for a conventional cC7 root transfer approach [6]. Third, the operative procedure is less technically challenging, since disruption of the cervical region can be avoided. Our modified approach also has biomechanical advantages, especially when compared to the modified method reported by Wang et al. [16]. In our modified procedure, a simple subcutaneous tunnel anterior to the neck and pectoral region in which the nerve passed through is made as it is always done in a conventional cC7 root transfer. According to Wang et al.’s experiences, the cC7 nerve was dissected up more proximally to the neuroforamina. The tip of a right-angle forceps was passed underneath the scalenus anterior muscle and the vertebral artery, and then it penetrated the longus colli muscle to the interval space between the carotid sheath and the esophagus on the donor side to create a tunnel. Next, one end of a plastic tube was drawn to initially pass through the tunnel. Then, its one end was drawn to the interval space passing through the retro-esophageal space on the recipient side. The cC7 root was wrapped in the plastic tube and sutured. Then, the cC7 root was passed to the affected side using the plastic tube as a guide.

Wang et al.’s [16] modified procedure, while superior in long-term outcomes compared to the conventional cC7 root transfer method, produced humerus shortening of 3 to 4.5 cm in 45.5% of the patients. This shortening was performed to allow the cC7 nerve to reach the lower trunk without a graft. This obviously could cause malfunction of the biceps muscle and triceps muscle. Our method, on the other hand, seems to have less risk for disturbing normal humerus length. Clinically, direct neurorrhaphy may increase tension at the anastomosis, leading to rupture of the anastomosis. In that case, it is much easier in our approach to identify the nerve suture by surgical re-exposure when it is in a subcutaneous region.

cC7 nerve transfer has not been widely adopted for several reasons. One is that there is a real danger of creating a deficit in the healthy donor limb during nerve harvesting. Furthermore, there is a controversy over the cutoff level of C7, and the literature regarding the appropriate length of the C7 root is lacking. To make full use of C7, many surgeons attempt to sever it at the level of the anterior division/lateral cord junction and posterior division/posterior cord junction [17]. A key step to avoid having to use nerve grafts as we describe here is to maximize the available length of cC7 by dissecting its anterior and posterior divisions as far distally as possible. By severing C7 at the division-cord level after opening the epineuria, the ADC7 and PDC7 are significantly longer (Fig. 6). According to a recent clinical study using a similar harvesting technique, no enduring deficit in the donor limb was observed during long-term follow-up [18]. Therefore, it may be safer and more reliable to harvest cC7 in this way.

It is clear that the degree of nerve fiber maturation, which is necessary to restore function, may depend partly on the distance of the lesion from any particular end organ. In the present study, the shortest distance between ADC7 and PDC7 and the medial epicondyle of the humerus along the grafting ulnar nerve was considered to be the nerve regeneration distance in conventional cC7 transfer. This mean distance turned out to be 44.8 cm, which was nearly 9 cm longer than the distance from the proximal ends of C8-T1 to the medial epicondyle of the humerus (mean = 35.3 cm). Thus, our method significantly shortens the nerve regeneration distance.

In conventional practice, nerve regeneration requires that axons traverse two neurorrhaphy sites. It is reasonable to conclude that this is not a favorable situation for optimal axon regeneration. Compared with earlier studies, direct cC7 transfer to the median nerve provided a remarkably superior outcome [16]. The authors concluded it was because of the shorter distance between the donor nerve and the recipient nerve, as well as having only one neurorrhaphy site. Similarly, there is only one neurorrhaphy site in the direct transfer cases we described in the present study, rather than two, as in conventional cC7 transfer. This situation engendered by our modified method theoretically facilitates axon passage through the nerve suture line to reach the muscles of the forearm.

It is well known that an insufficient number of myelinated fibers regenerating into the recipient nerves are a major factor leading to poor restoration of hand function after cC7 root transfer to the median nerve. First, the regenerating fibers grow only into the median nerve. Thus, the ulnar-dominated flexion muscles become beyond repair. By transferring cC7 to the C8-T1, both of them would be subject to re-innervation. Second, the grafting nerve or the recipient nerve is always narrower than the donor nerve, producing a mismatch and a waste of donor motor fibers. In the present study, we observed that the overall cross-sectional area of ADC7 and PDC7 matched very well that of C8 and T1 or lower trunk. In cases where C8-T1 of the affected limb is severely injured, fibrosis is present, or the nerve cannot be separated from the surrounding scar tissue, ADC7 and PDC7 still have a chance to be coapted with the lower trunk of the paralyzed brachial plexus.

The present study was not without limitations. The data were collected and evaluated from cadaver specimens fixed with formaldehyde. Thus, there might be some discrepancies in distances and measurements that would be measured in fresh corpses or in clinical investigations. Future clinical studies using larger sample sizes thus are necessary.

## Conclusions

Overall cross-sectional area of ADC7 and PDC7 matched very well that of C8 and T1 or lower trunk. For the treatment of tBPRA, a transfer of cC7 to the C8-T1 or lower trunk via a subcutaneous tunnel across the chest and neck appears to shorten the nerve graft and nerve regeneration distances compared with a conventional cC7 root transfer. Our modified method also decreases the number of neurorrhaphy sites and makes optimal use of donor nerves, which may benefit hand flexion restoration in clinical situations.

## Abbreviations

ADC7: Anterior division of C7
cC7: Contralateral C7 root
PDC7: Posterior division of C7
tBPRA: Total brachial plexus root avulsion

## References

[1] Gu YD, Zhang GM, Chen DS, Yan JG, Cheng XM, Chen L (1992). Seventh cervical nerve root transfer from the contralateral healthy side for treatment of brachial plexus root avulsion. J Hand Surg Br.

[2] Yang G, Chang KW, Chung KC (2015). A systematic review of contralateral C7 transfer for the treatment of traumatic brachial plexus injury: Part 1. Overall outcomes. Plast Reconstr Surg.

[3] Gu YD (2007). Contralateral C7 root transfer over the last 20 years in China. Chi Med J(Engl).

[4] Wang S, Yiu HW, Li P, Li Y, Wang H, Pan Y (2012). Contralateral C7 nerve root transfer to neurotize the upper trunk via a modified prespinal route in repair of brachial plexus avulsion injury. Microsurgery.

[5] Xu L, Gu Y, Xu J, Lin S, Chen L, Lu J (2008). Contralateral C7 transfer via the prespinal and retropharyngeal route to repair brachial plexus root avulsion: a preliminary report. Neurosurgery.

[6] Feng J, Wang T, Gu Y, Chen L, Zhang G, Zhu Y (2010). Contralateral C7 transfer to lower trunk via a subcutaneous tunnel across the anterior surface of chest and neck for total root avulsion of the brachial plexus: a preliminary report. Neurosurgery.

[7] Doshi PB, Bhatt YC (2016). Passage through the carotid sheath: an alternative path to the pre-spinal route for direct repair of contralateral C7 to the lower trunk in total brachial plexus root avulsion injury. Indian J Plast Surg.

[8] Moran SL, Steinmann SP, Shin AY (2005). Adult brachial plexus injuries: mechanism, patterns of injury, and physical diagnosis. Hand Clin.

[9] Zapalowicz K, Radek M. The distribution of brachial plexus lesions after experimental traction: a cadaveric study. J Neurosurg Spine. 2018;29:704-10. 10.3171/2018.5.SPINE171148 30265223

[10] Chuang DC (1999). Contralateral C7 transfer (CC-7T) for avulsion injury of the brachial plexus. Tech Hand Up Extrem Surg.

[11] Gao K, Lao J, Zhao X, Gu Y (2013). Outcome of contralateral C7 transfer to two recipient nerves in 22 patients with the total brachial plexus avulsion injury. Microsurgery.

[12] Sammer DM, Kircher MF, Bishop AT, Spinner RJ, Shin AY (2012). Hemi-contralateral C7 transfer in traumatic brachial plexus injuries: outcomes and complications. J Bone Joint Sur Am.

[13] Songcharoen P, Wongtrakul S, Mahaisavariya B, Spinner RJ (2001). Hemi-contralateral C7 transfer to median nerve in the treatment of root avulsion brachial plexus injury. J Hand Sur Am.

[14] Yu ZJ, Sui S, Yu S, Huang Y, Sheng J (2003). Contralateral normal C7 nerve transfer after upper arm shortening for the treatment of total root avulsion of the brachial plexus: a preliminary report. Plast Reconstr Surg.

[15] McGuiness CN, Kay SP (2002). The prespinal route in contralateral C7 nerve root transfer for brachial plexus avulsion injuries. J Hand Surg Br.

[16] Wang SF, Li PC, Xue YH, Yiu HW, Li YC, Wang HH (2013). Contralateral C7 nerve transfer with direct coaptation to restore lower trunk function after traumatic brachial plexus avulsion. J Bone Joint Sur Am.

[17] Qin BG, Fu G, Yang JT (2016). Microanatomy of the separable length of the C7. J Reconstr Microsurg.

[18] Bhatia A, Doshi P, Koul A, Shah V, Brown JM, Salama M (2017). Contralateral C-7 transfer: is direct repair really superior to grafting?. Neurosurg Focus.

